# IFEM executive summary white paper of climate and ecological crisis

**DOI:** 10.1007/s43678-024-00757-6

**Published:** 2024-08-07

**Authors:** Gayle Galletta, Lai Heng Foong, Simon Judkins, Alexander Robertson, Ffion Davies, Goma Bajaj, Constance LeBlanc, John Bonning, Faith Gaerlan, Wing Yee Clara Wu, Kwok Leung Tsui, Veronica Torres, Jonathan Kajjimu, Sarah Oworinawe, Roberta Petrino

**Affiliations:** 1 Department of Emergency Medicine, University of Massachusetts, Worcester, MA USA; 2 Department of Emergency Medicine, Bankstown Lidcomnbe Hospital, Sydney, NSW Australia; 3 Department of Emergency Medicine, Echuca Regional Health, Echuca, NSW Australia; 4 Department of Emergency Medicine, NHS Fife, Kirkcaldy, Scotland; 5 Department of Emergency Medicine, University Hospitals of Leicester, NHS, Leicester, England UK; 6 Department of Emergency Medicine, Shrimann Hospital, Jalandhar, Punjab India; 7 Department of Emergency Medicine, Dalhousie University, Halifax, NS Canada; 8 Department of Emergency Medicine, Hamilton, Waikato New Zealand; 9 Department of Emergency Medicine, University of the Philippines, Quezon City, Philippines; 10 Department of Emergency Medicine, Hong Kong College of Emergency Medicine, Hong Kong, China; 11 Hong Kong College of Emergency Medicine, Hong Kong, China; 12 Department of Emergency Medicine and Toxicology, Austral University Hospital, Buenos Aires, Argentina; 13 Department of Emergency Medicine, St. Francis Hospital-Nsambya, Kampala, Uganda; 14 Department of Emergency Medicine, Mbarara University of Science and Technology, Mbarara, Uganda; 15 Department of Emergency Medicine, Ente Ospedaliero Cantonale, Lugano, Switzerland

Healthcare plays a crucial role in society, but can also have unintended consequences on the environment, which can then negatively affect health. This is referred to as the environment–healthcare cycle. Healthcare has an enormous carbon footprint, contributing approximately 5% of global emissions, and is thus a major contributor to the climate and ecological crisis that our planet is now facing [1]. Climate change and its related disasters cause people to seek emergency medical care. Staff working in emergency departments must, therefore, be able to recognize and treat patients with climate-related conditions.

This white paper will review the effects of climate change on human health, the inequitable distribution of these negative effects, delineate how healthcare is harming the environment, and provide steps that can be taken to minimize healthcare’s carbon footprint. The full, unabridged white paper can be found on the International Federation for Emergency Medicine website: https://assets.nationbuilder.com/ifem/pages/905/attachments/original/1718599262/IFEM_White_Paper_on_Climate_and_Ecological_Crisis_June_2024.pdf?1718599262 [2].

## Background

The World Health Organization acknowledges that climate change is responsible for an increasing number and severity of public health emergencies including heatwaves, wildfires, hurricanes, and floods. These negative effects of climate change are not distributed equitably among individuals nor nations or regions. Developing countries with weak health infrastructure will be least able to cope with these public health emergencies. It is estimated that 3.6 billion people live in areas that are already highly susceptible to the effects of climate change, which will cause millions of additional deaths in the coming decades. Economically, the direct costs to health are estimated to be between US$ 2 and 4 billion annually by 2030 [3].

## The effect of the climate and ecological crisis on health

Climate change and increasing CO_2_ levels are causing global warming and increasing sea levels. This can affect health in many ways:

- Extreme heat: Heat waves occur more frequently and last longer, increasing direct heat illnesses such as heat stroke and all-cause mortality.
- Severe weather: Increasing frequency and intensity of superstorms, floods, and droughts lead to injury, death, destruction, unemployment, homelessness, and food and water insecurity, thereby disrupting the social and economic determinants of physical and mental health.
- Air pollution and allergens: Burning of fossil fuels and wildfire smoke worsen both asthma and cardiovascular disease, increasing morbidity and mortality, and emergency department (ED) visits [4].
- Changes in vector ecology and illness: increased infectious disease risk (malaria, dengue, chikungunya, West Nile Virus, Borrelia)
- Water pollution: water-borne illness (cholera, cryptosporidiosis, campylobacter, and leptospirosis)
- Water and food scarcity: crop failure, malnutrition, starvation, and mass migration causing population crowding in new areas
- Psychological trauma: Severe weather events often lead to civil conflict and forced migration, resulting in more severe mental health problems [5]. Mere awareness of the climate crisis is associated with emotional distress, especially in children and adolescents [6].

Researchers at the Massachusetts General Hospital in the United States found that weather disasters have a long-lasting effect on ED utilization and mortality, with utilization remaining elevated for two weeks and mortality remaining elevated for as much as 6 weeks after a weather disaster [7].

## Inequitable effect of climate and ecological crisis

The negative impacts of climate change are not equally distributed. The largest burden is borne by those living in the Global South, whereas the Global North has, in general, been responsible for the majority of climate change. Even within these two broad groups, it is the racially and ethnically minoritized groups, migrants, and Indigenous Peoples who are most negatively impacted by the health impacts of climate change [8].

Air pollution, one of the leading causes of death worldwide, has a more profound effect on low-income communities [9, 10]. Individuals at extremes of age or with pre-existing health conditions are also more susceptible to effects of climate change.

## The effect of healthcare on the environment

If healthcare were a nation, it would be the fifth largest emitter of greenhouse gases on the planet [11].

Healthcare’s contribution to greenhouse gas emissions (its carbon footprint) can be divided into three scopes [11]:

- Scope 1: Emissions under direct control of the healthcare facility (onsite energy production, fleet vehicles, and anesthetic gas leaks): 17% of carbon footprint.
- Scope 2: Electricity purchased by the healthcare facility: 12% of carbon footprint.
- Scope 3: Supply chain (production, transport, and disposal of medications, food, medical devices, and hospital equipment, employee commuting): 71% of carbon footprint.

Ten countries produce more than 68% of global greenhouse gas emissions. China leads the pack, followed by the US, India, and the European Union. However, if one looks at emissions per capita, it is Canada, the US, Qatar, and Australia that top the charts [12]. But, if one wants to determine healthcare’s share, one should rather look at how much each country spends on healthcare. According to the Organization for Economic Co-operation and Development (OECD), the average healthcare to Gross Domestic Product (GDP) ratio is 9%, with wide variations among countries. The US spends the most: 17.3% in 2022. In contrast, China spends only 5% of its GDP on healthcare [13].

Two types of medications, in particular, contribute to a large carbon footprint in health care: anesthetic gasses [14] and metered dose inhalers [15]. N_2_O is particularly harmful, with an atmospheric life of greater than 100 years. In addition, it damages the ozone layer and has 300 times the warming power of CO_2_ [14]. Metered dose inhalers (MDIs) use hydrofluorocarbon (HFC) as their propellant, which is also a powerful greenhouse gas. One hundred doses of a metered dose inhaler (MDI) are equivalent to driving a car 290 km [15].

Medical waste is a major contributor to healthcare’s carbon footprint. Single use, disposable medical equipment is estimated to comprise 85% of medical waste [16]. A large amount of food is also wasted [17]. A Portuguese study found that, on average, each patient throws away 953 g of food daily, representing 35% of the food served. Over the course of one year, this is estimated to cost 35.3 million Euros, or 0.5% of Portugal’s health budget [18]. Another area of waste is unnecessary testing and prescribing. Researchers in Canada and the UK studied the burden of unnecessary laboratory testing on patients, cost to the hospital, and harm to the environment (as measured by greenhouse gas emissions) [19]. In 2017, the Canadian Institute for Health Information estimated that 30% of healthcare provided that added no value for patients [20].

## Minimizing healthcare’s carbon and ecological footprint

We have a duty to not further contribute to climate change as this is harming our patients. Actions must be taken to mitigate the harmful effects of healthcare on the environment.

Some countries are implementing their own plans of action to meet the goals set forth by the Paris agreement. The Royal College of Emergency Medicine (RCEM) in the UK has developed the *GreenED* initiative “to measure and reduce the environmental impact of Emergency Departments in the UK, thus driving environmentally sustainable practices within the specialty of Emergency Medicine.” [21].

**Table:** 

Things *you*, as an EM physician, can do *today* to decrease healthcare’s carbon footprint
Create multidisciplinary sustainability workgroups at your hospital
Avoid single-use plastic and styrofoam cups and cutlery
Recycle appropriately
Use contaminated waste containers only as intended, not as normal waste
Turn off equipment, computers, and lighting when not in use
Use dry powder inhalers instead of MDIs
Avoid the use of N2O
Reduce the use of venipuncture and unnecessary testing
Avoid unnecessary glove use
Irrigate wounds with clean tap water instead of sterile water or saline
Minimize food waste, promote locally sourced food, and encourage a plant-based diet
Review discharge medications to eliminate excessive dispensing
Provide electronic rather than paper discharge instructions
Work remotely on non-clinical shifts and continue pandemic-inspired virtual meetings
Commute on foot, by bike, carpooling, or public transportation
Engage in advocacy, such as for affordable dry powder inhalers and other green initiatives
Sign the Academic Health Institutions’ Declaration on Planetary Health and commit to change. [22]

## IFEM’s role

IFEM is committed to advocating for better politically driven climate change policies and believes that emergency physicians have an influential part to play at global and local levels.

The Public and Environmental Health Special Interest Group (PEH-SIG) was founded in 2023. Our vision is to create a group within the international emergency care community that promotes best practice, education, research, advocacy, and collaboration about public and environmental health as it relates to emergency care [23].

IFEM will request that Local Organizing Committees pay attention to sustainability when planning the International Conferences on Emergency Medicine (ICEM). ICEM must also include sessions on climate change and sustainable healthcare, as climate change is the single greatest threat to planetary and human health. Furthermore, allowing a virtual option would enable members to participate in ICEM without increasing their carbon footprint through air travel.

IFEM supports Global EM Day, held on May 27 each year. The 2024 theme was “Climate Change is a Health Emergency, Too!” [24] Through global cooperation and education, IFEM accepts the challenge to improve planetary health, and therefore the health of our patients.

The healthcare sector has a responsibility to acknowledge and address the harmful effects that our practice has on the environment. By implementing mitigation strategies, together we can create a more sustainable healthcare system. As trusted voices in our communities, healthcare professionals have a large potential to influence societies’ support for action on climate change. There must be collaboration between healthcare providers, policymakers, and researchers to drive the positive change that is essential for a healthier future for both humans and the planet.
